# Meetings of Societies

**Published:** 1927

**Authors:** 


### Meetings of Societies.

### Bristol Medico-Chirurgical Society.

*January 12th,* 1927.

DR. A. L. FLEMMING, President, in the Chair.

The meeting was occupied by an address by MR. GREY TURNER, of Newcastle-upon-Tyne, on "**The Surgery of Malignant Disease.**" The purport of the address was that radical operation is still the best treatment for malignant disease, and that if practised optimistically it may afford many cures, so far as that term can ever be used in dealing with malignant disease, and often valuable improvement even in desperate cases. In support of this thesis, Mr. Grey Turner quoted a number of interesting and encouraging experiences from his own practice. The address was illustrated by a series of over 100 beautiful lantern slides, which served to support the lecturer's main argument.

*February 9th,* 1927.

DR. A. L. FLEMMING, President, in the Chair.

MR. A. E. ILES opened a discussion on "**The Prophylaxis of Ophthalmia Neonatorum,**" an abstract of his paper appearing on page 125.

In the discussion which followed, THE PRESIDENT said that fifteen years ago 50 per cent. of the children at the Blind School were totally blind ; to-day the proportion was 10 per cent. only, and the proportion of severe blindness similarly less. He asked how soon after the onset of ophthalmia were the cases brought for treatment.

MR. E. R. CHAMBERS agreed that mercurochrome was a valuable remedy. He still felt, however, that for gonococcal infections silver nitrate was the best prophylactic. The necrosis of the superficial epithelium which it causes was of value, and one should be slow to abandon this remedy, the worth of which was established. Frequent irrigation is an important part of treatment of the established disease. Two per cent. silver nitrate used as an eye-drop, instead of as a paint for the lids, will cause severe burns. He preferred to neutralise the silver solution with saline after each application. External canthotomy was a very valuable adjunct to treatment.

MR. CYRIL H. WALKER said that irrigation of the eye of a small baby is very difficult, but is exceedingly valuable and indeed often sufficient treatment, without the addition of any disinfectants. But sometimes it fails and silver nitrate becomes necessary. He did not neutralise the latter, as it was sufficient to remove excess. He had never seen any harm from occasional use, but bad damage to the cornea and even to the lid may occur if it is used repeatedly. Formaldehyde was at one time, he remembered, in fashion, on account of its penetrating power, but it was painful. From the point of view of reaction mercurochrome had a great advantage in treatment over silver, and further it was easy to be sure that it had got right into the eye. Perhaps in treatment we had been too energetic with silver, which could certainly do damage in the late stages. But the tissue stimulation was of service in provoking the normal defensive mechanism of the eye. He had no faith in colloidal silver.

DR. R. S. STATHAM said that just after the war he was using weak boric lotion only, but found the method unsatisfactory. Silver nitrate was very efficient, and on the Royal Infirmary "District" its use abolished ophthalmia. He felt quite sure, however, that the midwives generally of the city did not use it. Prophylaxis was the most important element in treatment, and treatment of gonorrhœa in the expectant mother was essential. Mercurochrome was ideal for cervicitis.

DR. D. A. ALEXANDER doubted whether there was any value in colloidal silver. He advised the use in general practice of mercurochrome pessaries. He asked how soon one should look for a return of ophthalmia after apparent cure.

MR. ILES, in reply, defended the claims of mercurochrome ; it was a specific for the gonococcus, and had great powers of penetration. In conjunctivitis he agreed that frequent irrigation was very valuable, and he preferred argyrol to nitrate. He was conscious that prophylaxis was not generally or properly carried out by the independent midwives of the city. He advised watching for four weeks after the cessation of treatment before letting the patient disappear.

MR. E. WATSON-WILLIAMS then read a paper on "**The Significance of Vertigo,**" published on page 119 in this issue.

In the discussion which followed MR. A. J. WRIGHT said that he agreed that many cases of vertigo had some aural lesion, but there was usually deafness or tinnitus in addition to vertigo. He thought vertigo was not common in catarrhal otitis, though it was frequently seen in otosclerosis, where the labyrinth itself was invaded. He went on to speak of the question of localisation of the lesion. Often it was impossible to distinguish labyrinthine from intracranial disease. If there was deafness to high notes, this would be in favour of a labyrinth lesion. In regard to treatment he thought that a very important point was to reassure the patient, to convince him that he was not suffering from some serious or fatal disease, and that the condition would tend to disappear, though it might be long in so doing He deprecated local treatment.

DR. P. WATSON-WILLIAMS pointed out that operative treatment of the ear was seldom required. The importance of treating other parts, disease in which might affect the ears, should not be forgotten. For example, an infection of the nasal sinuses might be at the root of the trouble. He believed that the significance of morning anorexia in these cases was a new observation.

DR. CARLETON said that he had been interested in the vertigo that occurred in encephalitis lethargica. He considered that the middle ear was one portal of entry of the virus, and had found that in this disease there were frequently disturbances of the vestibular reactions.

THE PRESIDENT inquired how Mr. Watson-Williams would explain the occurrence of endemic paralytic vertigo.

MR. E. R. CHAMBERS said that disorder of the eye muscle balance frequently produced very severe giddiness. He instanced a recent case relieved by wearing a prism.

DR. D. A. ALEXANDER was interested in the anorexia of aural disease. He mentioned a case in which excessive appetite was apparently connected with otitis media.

MR. E. WATSON-WILLIAMS, in reply, said that he had avoided all questions of diagnosis and treatment; his purpose was to draw attention to the localising significance of true rotatory vertigo. Endemic or epidemic paroxysmal vertigo was seen in the eighties and nineties of last century in Switzerland. It occurred only in summer, and affected those who worked in stables; cats were also attacked. The same disease had been seen in Japan. There were other phenomena, unconsciousness, paralysis, and laryngeal spasm; one must consider this a disease of the central nervous system. It was never fatal. With diplopia some degree of dizziness or uncertainty was common, but he believed that a rotatory vertigo only occurred when the ocular paralysis was due to intracranial disease.

---

*March 9th*, 1927.

DR. A. L. FLEMMING, President, in the Chair.

**"The Treatment of Cancer."**

DR. TODD opened his paper by a brief glance at the methods available for the production of colloidal lead. He rejected electrical methods owing to the variable nature of the product. Partly, perhaps, on account of the work done in Bristol during the last seven years with selenium, the value of that substance in the treatment of cancer was locally well recognised. It occurred, therefore, to him to attempt to combine lead with selenium, so as to make use of the analgesic properties of the latter while employing the former, and in the summer of 1926 a colloid of lead selenide was successfully produced by chemical methods. On the hypothesis which Blair Bell had adopted, the resemblance of growth in a cancer to that of the chorion indicated that a drug likely to arrest the one should destroy the other; in other words, a cancer cure should be a good abortifacient. Dr. Todd, at this point, showed slides illustrating the effect of his first lead selenide on the rabbit's placenta, on the cells of the liver and on the blood; in the latter, changes in types of the cells were produced as well as a secondary anæmia. A series of compounds was prepared, tested and one had been tried on human subjects of inoperable cancer, to test the hypothesis. A palliative effect only was expected. Three main factors governed the effect :—

(a) The rapidity of the growth and its avidity for lead.
(b) The size of the malignant mass.
c) The ability of other vital tissues to survive a dose fatal to the cancer.

The more malignant a tissue, the greater was its lipoid content and its resemblance to the decidua ; a slow-growing cancer was probably less suitable for treatment on these lines. A table was given illustrating the first of these points. Dr. Todd mentioned that the colloid he was working with was found to be less selective than had been hoped : its dosage was still in the experimental stage. He described the very great care necessary, not only in preparing and standardising the drug, but in investigating and supervising the condition of the patient. It was most essential to deal carefully with even small septic foci. The hepatic and renal functions had to be thoroughly tested. Ultra-violet light baths were used to combat the anæmia, small doses of magnesium sulphate to prevent colic and, between courses of treatment, potassium iodide to set free lead " fixed " in the skeleton. The dose he was using ranged from 40 to 100 mgm. of lead at a dose, and he hoped to give $1\frac{1}{2}$ gm. of lead a year in three courses in favourable cases. Dr. Todd then discussed the immediate symptoms which might follow an injection, which he thought due to its colloidal nature, and some of the late results, which he ascribed to lead poisoning : among the latter great mental depression was observed, colic was rare and mild, possibly owing to the presence of selenium, and in the treatment of colic adrenalin had been found useful elsewhere. The author then briefly reviewed reports of twelve cases treated by this method. He concluded that while lead selenide has a definitely regressive action on cancer, the treatment was still in the experimental stage ; the results were not yet ready for publication nor was the method generally available. In conclusion, he hoped that nobody would go away and say that there had been discovered a new cure for cancer, which would be both unfair and untrue.

In the discussion which followed MR. E. WATSON-WILLIAMS described the case of carcinoma of the gullet which was treated first with collosol lead, then with Dr. Todd's colloid of lead selenide. The first preparation produced no effect on the growth, though a distinct "blue line" was evidence of absorption of lead; the second was much more potent, producing marked general changes and leading to disappearance of the growth, and its replacement by a firm, fibrous scar. The situation prevented any more minute examination; but while the patient was waiting for the second course he developed dysphagia, and this was found to be due to another growth, about 7 cm. above the first. He died soon after, at Christmas, 1926, but no autopsy was allowed. Another case received too small a dose to be of value, and would not continue. Later, Mr. Watson-Williams said that when working with selenium he found, first, that the carcinoma of columnar cell tubes, such as stomach or uterus, was more easily affected than that of squamous-lined cavities, such as the pharynx; secondly, that primary growths were more susceptible than secondaries; and, thirdly, that relief of pain and cessation of discharges were the most striking features, even where the relief was only temporary.

Mr. A. R. Short described a very hopeless case of cancer of the tongue that Dr. Todd had treated for him. He thought there was some discomfort from the lead injections, but the patient was definitely better for the treatment; this was not completed, and the patient was now dead.

Dr. R. S. Statham stated that he had had five cases of carcinoma of the uterus treated, and was very enthusiastic at the results. The improvement was dramatic, and it had been found possible to overcome the mental repugnance to the injections by keeping the patients under morphia. The first case had become operable, from being quite hopeless, ulceration having disappeared. The second case showed a growth which became replaced by a mass of scar tissue, and pain vanished. The same good result followed with Nos. 3 and 4. Case 5 was a malignant papilliferous ovarian cyst. The whole peritoneum was affected. After treatment the growth became much less, but owing to shrinking some intestinal obstruction developed. Laparatomy was necessary for intestinal anastomosis, and it was then found that the growth was represented by a small hard lump only, and the ascites had practically disappeared. There were no such massive adhesions as follow the use of X-rays.

Mr. A. W. Adams said a case of his with cancer of the lip had been treated; it became cleaner, but the patient did not react well.

MR. JACKMAN related how a very large and fixed cancer of the breast had become small; he had removed the growth and breast complete, and showed a photograph of the condition. It was from his case that Dr. Todd had had sections showing the effect on the growth of the treatment.

DR. EDGEWORTH mentioned a case he had treated with selenium. The growth was situated in the head of the pancreas, and before treatment presented a palpable tumour. This quite disappeared, and the accompanying jaundice became very much better. The patient died later of hæmatemesis. He thought there was great relief from pain.

THE PRESIDENT (Dr. A. L. Flemming) asked whether it was possible to employ the injection locally in early cases.

DR. G. B. BUSH asked whether there was evidence that lead was deposited in the tumour. If so, it was worth considering whether one might employ X-rays after the course of injections, hoping to get secondary radiations from the metal.

DR. ALEXANDER asked why alimentary administration of lead should not be as efficacious as injection. It certainly acted as an abortifacient when thus administered. He had used the pil. plumbi c. opio for rectal carcinoma with gratifying results.

DR. TODD, in reply, said that it was possible that the early stages of cancer were less amenable to treatment in this way than the later, as being composed of less malignant tissue. There would be less phosphatid content in such growths, and it was probable that this is what determined the affinity of malignant tissue for lead. The effect on metastases was less than on the primary growths, and he thought the alimentary and genital types of epithelium reacted best. Lead by the mouth had a definite inhibitory effect, but this was slight; cancer could occur in a victim of lead poisoning.

